# Case report: Dynamic antibody monitoring in a case of anti-recombinant human erythropoietin-mediated pure red cell aplasia with prolonged course after kidney transplantation

**DOI:** 10.3389/fimmu.2022.1049444

**Published:** 2022-11-29

**Authors:** Xiao-Mei Chen, Hui Li, Yu Wu, Lan-Lan Wang, Yang-Juan Bai, Yun-Ying Shi

**Affiliations:** ^1^ Department of Laboratory Medicine/Research Centre of Clinical Laboratory Medicine, West China Hospital, Sichuan University, Chengdu, Sichuan, China; ^2^ Department of Nephrology, West China Hospital, Sichuan University, Chengdu, Sichuan, China; ^3^ Department of Hematology, West China Hospital, Sichuan University, Chengdu, Sichuan, China

**Keywords:** anti-erythropoietin (anti-EPO) antibody, pure red cell aplasia, kidney transplant, immunosuppressive therapy, roxadustat

## Abstract

Anti-erythropoietin (anti-EPO) antibody-mediated pure red cell aplasia (PRCA) is a rarely seen disease. Anti-EPO antibodies were mostly found in patients with chronic kidney disease who received recombinant human erythropoietin (rHuEPO) injections subcutaneously. The treatment against anti-EPO antibody-mediated PRCA included discontinuation of rHuEPO, immunosuppressive agents, intravenous immunoglobulin, plasmapheresis, or kidney transplantation. We reported a case of kidney transplant recipient with anti-EPO antibody-mediated PRCA, who had no trend of recovery after stopping rHuEPO, receiving regular induction and maintenance immunosuppressive regimens. He was further given 6 consecutive plasmapheresis sessions, cyclophosphamide, and adjusted maintenance immunosuppressive regimen into cyclosporine, sirolimus and prednisone. We monitored his anti-EPO antibody levels with a self-created simple mixing test. At 10 months post kidney transplant, his anti-EPO antibody finally turned negative, and his reticulocyte count dramatically increased. Cyclosporine, sirolimus and prednisone combined with roxadustat eventually alleviated the patient’s anti-EPO antibody-mediated PRCA. Our self-created simple mixing test for anti-EPO antibody titer was very helpful in disease monitoring and therapeutic guidance.

## Introduction

Anti-EPO antibody-mediated PRCA is a very rare but severe transfusion-dependent anemia with an incidence of 0.02 to 0.03 per 1000 person-years (1). The incidence rate may be underestimated due to the availability of anti-EPO antibody testing. A slight modification in the production process of rHuEPO leads to some antigenicity of the manufactured hormone, which induces the generation of anti-EPO antibody (2–4). Causes of this disease included formulations without human serum albumin, subcutaneous administration, and uncoated rubber stoppers (1). The median duration of rHuEPO treatment prior to the occurrence of PRCA was 9-25 months (5). There was no guideline on the treatment for anti-EPO antibody-mediated PRCA, because there were too limited cases to perform prospective cohort studies and the patients in most case reports experienced rapid remission after kidney transplant (6). Since kidney transplant itself was an effective treatment for anti-EPO antibody-mediated PRCA, cases with prolonged course after kidney transplant were rarely reported. We reported a case of anti-EPO antibody-mediated PRCA diagnosed after kidney transplant with an abnormally prolonged course, and we successfully created a simple mixing test to monitor anti-EPO antibody titer and guide our treatment adjustment effectively.

## Case presentation

**Figure 1:**
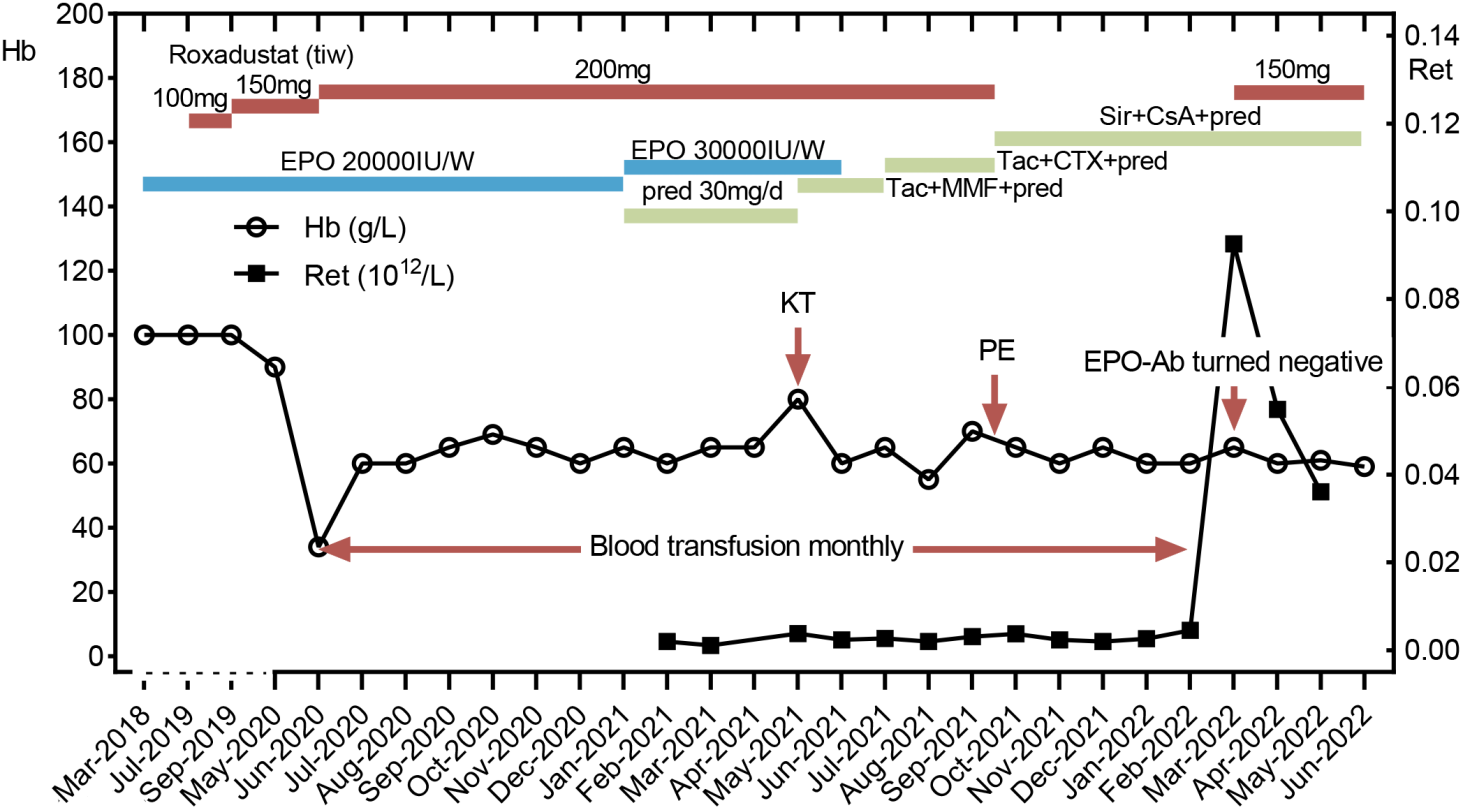
Clinical course. A kidney transplantation was performed in May 2021. Plasma exchange was performed in September 15, 2021. After the EPO antibody turned negative, the patient didn’t rely on blood transfusion any more. Hb, hemoglobin; Ret, reticulocyte; Tac, tacrolimus; MMF, mycophenolate mofetil; pred, prednisone; CTX, cyclophosphamide; Sir, sirolimus; CsA, cyclosporine; KT, kidney transplant; PE, plasma exchange.

A 38-year-old Chinese man who was diagnosed with end-stage renal disease (ESRD) due to chronic glomerulonephritis started maintenance hemodialysis three times a week in a local hospital since 2018. He received rHuEPO (Epiao, 3SBio, Shenyang, China) subcutaneously at 10,000 IU twice a week and roxadustat was added later due to his hemoglobin (Hb) below the target range (**Figure 1**). In June 2020, his Hb level suddenly decreased from 100 g/L to 34 g/L without evidence of active bleeding or hemolysis, and he required blood transfusion every month to maintain Hb around 60g/L ever since then. He underwent his first bone marrow aspirate and biopsy in February 2021 in West China Hospital, and his bone marrow smear showed hypercellularity with no red blood cell precursors. His blood routine examination showed that reticulocyte count was 0.0020×10^12^/L and Hb was 50 g/L. His erythropoietin level was <0.60 mIU/mL and his ferritin level was >2000 ng/mL. In May 2021, he received a kidney transplant donated by his 58-year-old mother in West China Hospital of Sichuan University, and his Hb was enhanced to 90 g/L by transfusing leukodepleted red cell suspension prior to the surgery. Induction therapy including intravenous basiliximab and methylprednisolone pulse therapy was given, and the standard triple immunosuppressive regimen consisting of mycophenolate mofetil (MMF), tacrolimus (Tac), and prednisone was immediately applied. Trimethoprim-sulfamethoxazole and ganciclovir were administered as the general prophylaxis for pneumocystis pneumonia and cytomegalovirus (CMV) infection, respectively. The kidney graft functioned immediately after the surgery, and roxadustat combined with rHuEPO injection subcutaneously were continued for his anemia (**Figure 1**).

Approximately one month after kidney transplant, the patient was readmitted to the hospital due to severe anemia. His blood routine examination showed that reticulocyte count was 0.0020×10^12^/L, Hb was 49 g/L, platelet (PLT) count was 60×10^9^/L and white blood cell (WBC) count was 2.8×10^9^/L with normal differentials. His graft function was stable with a creatinine level of 144 µmol/L. The erythropoietin and ferritin levels were similar to those before the transplant. Examinations to exclude other possible causes of anemia, including tumor markers, serum protein electrophoresis, serum immunofixation electrophoresis, anti-nuclear antibodies (ANA), extractable nuclear antigens (ENA), Coombs test, serum cytomegalovirus (CMV) DNA polymerase chain reaction (PCR) and Epstein-Barr virus (EBV) DNA PCR, were all negative. Computed tomography of the chest did not reveal thymoma. Fecal occult blood testing and DNA quantification of parvovirus B19 were also negative. No suspected family history or history of using drugs that might interfere hematopoietic function was found. Tacrolimus trough concentration was 6.86ng/mL. A second bone marrow biopsy was performed, and the bone marrow smear showed hypercellularity with 0.5% red blood cell precursors. Bone marrow biopsy revealed severe erythroid hypoplasia. The patient was diagnosed with PRCA accordingly. His serum was sent to 3SBio Pharmaceutical Company for the anti-EPO antibody examination, which showed positive by enzyme-linked immunosorbent assay (ELISA). The neutralization test of anti-EPO antibody was performed by a bioassay based on the fact that rHuEPO-neutralizing antibodies could inhibit the proliferation of rHuEPO on UT7/EPO-dependent cell lines. The patient was eventually diagnosed with anti-EPO antibody-mediated PRCA.

**Figure 2:**
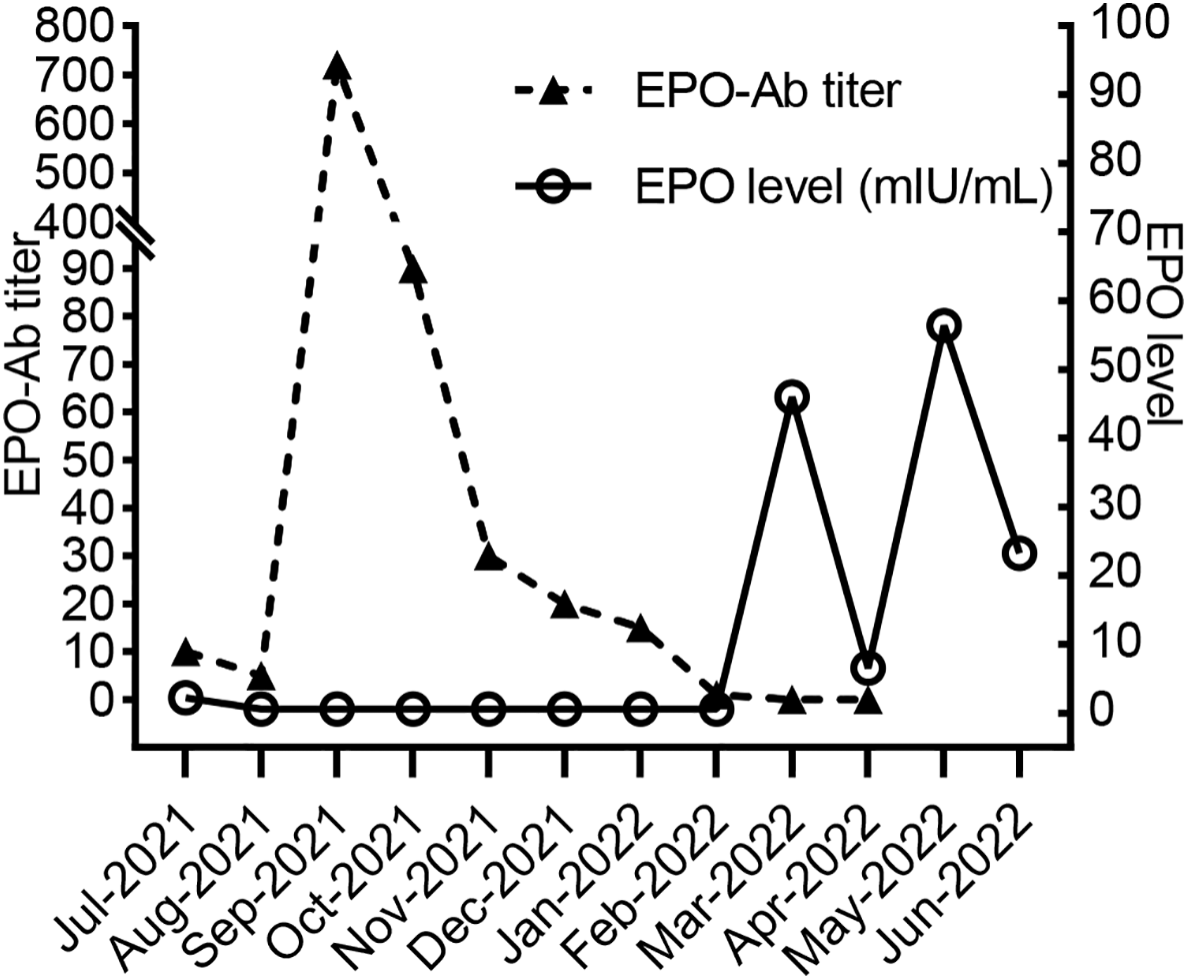
EPO antibody titer and EPO levels of the patient.

Due to the limited availability and long turn-around time of the anti-EPO antibody test, as well as the need for dynamic monitoring of the therapeutic effect, we created a simple mixing test to quantify the antibody titer (**Supplementary Figure 1**). The operating steps were as follows: the sera of both the patient and the health control were collected and then mixed in different proportions, which were used for the testing of EPO concentrations (IMMULITE 1000, SIEMENS). The maximum dilution multiple at which a positive EPO result was obtained was defined as the antibody titer, which was based on the fact that the neutralizing IgG antibodies against the protein component of exogenous erythropoiesis-stimulating agents (ESAs) would cross-react with endogenous hormones. The result of the simple mixing test was consistent with the neutralization test of anti-EPO antibodies from the 3SBio Pharmaceutical Company, which revealed a titer of 1:10 at diagnosis. Then, we adopted this simple method for the monthly anti-EPO antibody monitoring. It should be noted that the EPO level of the selected health control in each test must be fixed and above the upper limit of normal value to reduce errors. In our experiment, the fixed EPO level was 200mIU/ml. The dynamic changes of anti-EPO antibody titers and EPO levels of the patient are shown in **Figure 2**.

Once diagnosed in July 2021, rHuEPO was stopped immediately. The detailed treatment course is shown in **Figure 1**. Recombinant human granulocyte colony-stimulating factors were administered and Trimethoprim-sulfamethoxazole as well as ganciclovir were discontinued due to leukopenia. The antibody titer decreased to 1:5 in August 2021, and then suddenly increased to 1:720 one month later after he received massive transfusion of 10 U leukodepleted red blood cell suspension in the local hospital. It was speculated that EPO existed in a small amount of plasma contained in the leukodepleted red blood cells, which may activate immunological memory response and induce the massive production of anti-EPO antibodies. In comparison, the residual plasma volume in washed red blood cells was less than that in leukodepleted red blood cell suspension (7). Therefore, we replaced the leukodepleted red blood cell suspension with a restrictive washed red blood cell transfusion. At the same time, the cumulative dose of cyclophosphamide reached 6 g but no effect was observed. Then, we performed plasmapheresis 6 times and adjusted immunosuppressive regimen into cyclosporine, sirolimus and methylprednisolone (because of mild liver dysfunction). The trough concentration of cyclosporine was maintained at 100-150 ng/ml, and sirolimus was maintained at 6-8 ng/ml. By the fifth month of the above treatment, antibodies were finally undetectable using the simple mixing test, and laboratory results were as follows: a Hb level of 51 g/L, a reticulocyte count of 0.0926×10^12^/L, an EPO level of 46 mIU/mL, a creatinine level of 146 µmol/L, and an eGFR of 51.46 mL/min/1.73 m^2^. A serum sample of the patient was sent to 3SBio Pharmaceutical company again for anti-EPO antibody test, which also confirmed the negative result. We did not schedule any more blood transfusions but started roxadustat treatment. For the following three months, although the patient’s hemoglobin still fluctuated around 60 g/L, he no longer relied on blood transfusions. During the whole treatment, the graft function of the patient remained stable with serum creatinine level around 120-140umol/L, and no acute rejection or severe infection was occurred except a mild urinary tract infection and a herpes zoster infection.

## Discussion

The main treatments for anti-EPO antibody-mediated PRCA include immediate cessation of rHuEPO, restrictive transfusion and immunosuppressive therapies (8). However, long-term blood transfusions are resource-consuming and may increase the risk of infection and antibody development. Kidney transplant appears to be a viable option for ESRD patients with PRCA (9). There may be antigenic differences between endogenous and exogenous erythropoietin, and kidney transplantation can inhibit the production of antibodies and restore EPO secretion from the transplanted kidney (10, 11). A retrospective study collected anti-EPO antibody-mediated PRCA cases in French and German from 1998 to 2003, which found that there were 6 cases in total and they all got recovered one month after undergoing kidney transplant (6). We also reviewed other case reports published afterward and found that 6 (85.7%) out of 7 ESRD cases with anti-EPO antibody-mediated PRCA got recovered with Hb levels above 100 g/L within 3 months after kidney transplantation (12–15). Only one case reported partial recovery at 28 months after transplantation with persistent anti-EPO antibodies but independent of blood transfusion (13). The clinical characteristics of 13 reported cases of anti-rHuEPO antibody-mediated PRCA who underwent kidney transplant were summarized in **Table 1**. Unlike previous reports, anemia in our case did not recover quickly after kidney transplant and intense immunosuppressive regimen. Instead, the antibodies disappeared after plasmapheresis therapy and immunosuppressive regimen adjustment. Although the anemia in this case was not completely corrected, we still consider the treatment to be successful because of the negative antibody result, increased reticulocyte count and detectable EPO concentration.

**Table 1:** Clinical characteristics of 13 reported cases of anti-rHuEPO antibody-mediated PRCA who underwent kidney transplantation.

Case reports	Age(y)/sex	Primary disease	Forms of rHuEPO given	Time from rHuEPO treatment to PRCA occurrence	Mean weekly dose	Diag-nosis before KT	Treatment before KT for PRCA	Induction therapy	Donor type	Immunosu-ppressive regimens after KT	Outcome		
											Hb (g/L)	EPO-Ab	Time from KT to recovery
Verhelst et al., 2004 (6) ^*^	NA	NA	NA	NA	NA	Yes	NA	IL-2 receptor antagonists or antilymphocyte globulins	NA	Tac or CsA, MMF, steroid	NA	NA	within 1 month
Kitpermkiat et al., 2022 (12)	46/F	LN	CERA, Biosimilar Retacrit^®^ (alpha)	21 months	7200 IU	Yes	None	Rituximab, DFPP, IVIG, ATG, Tac, MMF, steroid,	living-related donor (brother)	Tac, MMF, steroid	127	NA	3 months
Nigg et al., 2004 (13)	25/F	MPGN	Eprex^®^ (alpha), Recormon^®^ (beta)	9 months	7500-12000 IU; 7000-20000 IU	Yes	CsA, CTX, steroids, ATG, thymectomy	NA	living-related donor (sister)	CsA, MMF, steroid	63-75	positive	28 months (partial remission)
Snanoudj et al., 2004 (14)	15/M	drug-induced (cyclosporine)	Eprex^®^ (alpha), Neorecormon^®^ (beta)	10 months	2000-8000 IU	Yes	CsA, AZA, steroid for liver transplantation	ATG, steroid	cadaveric donor	Tac, MMF, steroid	120	negative	6 weeks
Praditpornsilpa et al., 2005 (15)	15/NA	NA	alpha and beta rHuEPO	30 months	267 IU/kg	Yes	Decadurabolin, steroid, CTX	Daclizumab	cadaveric donor	CsA, AZA, steroid	over 100	negative	12 weeks
	44/NA	NA	beta and alpha rHuEPO	14 months	267 IU/kg	Yes	None	Daclizumab	cadaveric donor	CsA, AZA, steroid	over 100	negative	8 weeks
	52/NA	NA	beta rHuEPO	22 months	267 IU/kg	Yes	None	Daclizumab	cadaveric donor	CsA, AZA, steroid	over 100	negative	12 weeks
	53/NA	NA	alpha rHuEPO	26 months	267 IU/kg	Yes	None	Daclizumab	cadaveric donor	CsA, AZA, steroid	over 100	negative	10 weeks

^*^The retrospective study concluded 6 cases in total. rHuEPO, recombinant human erythropoietin; PRCA, pure red cell aplasia; KT, kidney transplant; EPO-Ab, anti-EPO antibody; LN, lupus nephritis; CERA, continuous erythropoietin receptor activator; Tac, tacrolimus; MMF, mycophenolate mofetil; DFPP, double-filtration plasmapheresis; IVIG, intravenous immunoglobulin; ATG, anti-thymocyte globulin; MPGN, membranoproliferative glomerulonephritis; CsA, cyclosporin A; CTX, cyclophosphamide; AZA, azathioprine; NA, data not available.

The patient was diagnosed 1 month after kidney transplantation after identifying anti-EPO antibodies, which highlights the importance of the timely detection of anti-EPO antibodies in suspected patients (16). The timely diagnosis before renal transplant can not only prevent the accumulation of rHuEPO but also guide the selection of immunosuppressive agents for subsequent treatment. Current assays for anti-EPO antibody examination include radioimmunoprecipitation (RIP), enzyme linked immunosorbent assay (ELISA), surface plasmon resonance (BIAcore) and bioassays that measure the proliferation of EPO-dependent primary erythroid cells or cell lines. Characteristics of the four assays used to measure anti-EPO antibodies are shown in **Table 2**. No single assay can both detect and fully characterize the presence of Abs and determine their neutralizing capabilities of them. Each assay has its own particular level of sensitivity and specificity for detecting Ab isotypes or binding affinities. At least two assays must be used for the analysis of EPO Abs, one assay for confirming the existence of Abs and the other (a bioassay) to demonstrate the Abs’ ability to inhibit the biological activity of epoetin in living cells. Various laboratories have used different assays for detecting anti-EPO Abs. Due to the lack of standardized processes and reagents, it is difficult to compare the test results from different laboratories directly. In addition, laboratories capable of carrying out such tests were quite few, which leads to the extremely long turn-around-time for the anti-EPO antibody detection. Only a few previous cases have reported antibody titer monitoring during treatment with sophisticated bioassay methods. We recommended the aforementioned simple mixing test for the diagnosis and therapeutic monitoring of PRCA mediated by anti-EPO antibodies. Our simple mixing test is time-and cost-effective, which can be used for rapid differential diagnosis and timely therapeutic effect evaluation.

**Table 2:** Characteristics of the four assays used to measure anti-EPO antibodies.

Reference	Assays	Detection principles	Type of Anti-EPO antibodies detected	Lower limit of sensitivity	Advantages	Disadvantages	Measures Ab isotypes	Measures Ab affinities
Swanson et al., 2004 (17)	ELISA (conventional)	Indirect enzyme-linked immunosorbent assay	IgG	78IU/mL	Short detection time	Low affinity antibodies may not be able to detect	Yes, in some cases	No
					Relatively easy to use in the laboratory	Relatively low specificity and sensitivity		
					Detection reagents and instruments are relatively cheap	Unable to detect anti-EPO antibody IgM Not conducive to early diagnosis of disease		
					High throughput analysis - commonly used as a “screening assay” for anti EPO antibody detection			
Shin et al., 2012 (18)	ELISA (bridging)	Bridging enzyme-linked immunosorbent assay	IgG	40IU/mL	Dual-arm binding improves specificity (anti-EPO antibody needs to be recognized twice/to be detected)	Requires expensive enzyme-labeled antigens	Yes, in some cases	No
						Unable to detect anti-EPO antibody IgM		
Casadevall et al., 2002 (19)	RIP	Radioimmunoprecipitation assay	IgG	0.2IU/mL	High sensitivity and specificity	Difficult to automate; low-throughput analysis	No	No
					High throughput analysis	Risk of radionuclide contamination		
						Low affinity antibodies may not be able to detect		
						Difficult to detect anti-EPO IgM antibodies		
Swanson et al., 2004 (17)	BIAcore	Biosensor Immunoassay, surface plasmon resonance	IgG, IgM, subtype of antibody	8-10IU/mL	Detects the concentration of anti-EPO antibody, measures the affinity of the antibody, and also determines the subtype of the antibody	Expensive special testing equipment Available only in a few high-tech laboratories	Yes	No
					Can detect “low affinity” antibodies	Antigen degradation may result in false negatives during the procedure		
					High specificity	Less sensitivity		
Casadevall et al., 2002 (19)	Bioassay	*In vitro* bioassays that measure proliferation of EPO-dependent primary erythroid cells or cell lines	Not mentioned	1IU/mL	Functional analysis to differentiate antibodies with neutralizing potential The only test that demonstrates the ability of antibodies to neutralize endogenous EPO	Complicated operation and time-consuming Validation difficult	No	Yes

In this case, a large number of antibodies were removed from a titer of 1:720 to 1:30 after 6 rounds of plasmapheresis. To protect the graft kidney function, preventing the production of EPO-neutralizing antibodies and eliminating residual antibodies is essential. Therefore, we finally chose cyclosporine, sirolimus and prednisone for immunosuppressive treatment. After four-month treatment of the adjusted regimen, the antibody titer dropped to 1:1, the reticulocyte count began to rise, and the transfusion frequency began to drop. At the fifth month of the treatment, EPO could be detected in the patient’s serum, and the reticulocyte count was higher than the normal range, indicating that the bone marrow began to restore hematopoiesis. To our knowledge, this was the first reported case of successful remission of persistent PRCA after kidney transplantation using such treatment regimen. Moreover, this patient’s PRCA was refractory with an abnormally prolonged disease course. Recently, studies had recommended initial treatment, including cyclosporine or cyclophosphamide combined with prednisone in PRCA (9, 11, 20). Tacrolimus may be considered as a substitute for cyclosporine (21). Chen et al. also reported the therapeutic effect of sirolimus in PRCA patients with a complete response of 58.3% and a median time of 4 (1–7) months to achieve the optimal effect (22).

In addition, roxadustat is an oral hypoxia-inducible factor prolyl hydroxylase inhibitor that simulates intracellular hypoxia to promote the production of endogenous EPO. Successful treatment with roxadustat in anti-EPO antibody-mediated PRCA has been noted in some case reports (23–25). However, it was reported that anti-EPO antibodies were found in patients who had never received rHuEPO (26), indicating that endogenous EPO may also induce the development of autoantibodies. Therefore, considering the possible interference of roxadustat in antibody production and its limited therapeutic effect when massive anti-EPO antibodies still existed, we stopped roxadustat treatment when anti-EPO antibody titer increased dramatically in Sep 2021 and restarted roxadustat when anti-EPO antibodies were below the lower limit of detection in Mar 2022. Our result demonstrated the safety of roxadustat in PRCA because no reproduction of anti-EPO antibody was found after restarting roxadustat. However, the data of our patient did not show the effectiveness of roxadustat, which may be related to the short observation time.

In conclusion, we reported a case of anti-EPO antibody-mediated persistent PRCA after renal transplant. Cyclosporine, sirolimus and methylprednisolone could be considered as maintenance immunosuppressive regimen and washed red blood cells should be used instead of leukodepleted red blood cells if transfusion was needed. Plasmapheresis was useful when anti-EPO antibody titers reached quite high. We created a simple mixing test for anti-EPO antibody titer, which was helpful in dynamic antibody monitoring especially when the examination of anti-EPO antibodies with RIPA, ELISA or biosensor assay was not available.

## Data availability statement

The raw data supporting the conclusions of this article will be made available by the authors, without undue reservation.

## Ethics statement

The studies involving human participants were reviewed and approved by Committee on Medical Ethics of West China Hospital, Sichuan University. The patients/participants provided their written informed consent to participate in this study. Written informed consent was obtained from the individual(s) for the publication of any potentially identifiable images or data included in this article.

## Author contributions

The original manuscript was written by HL and X-MC and reviewed by Y-YS, Y-JB, L-LW and YW, while YW and Y-YS participated in the treatments for the patients. The test for anti-EPO antibodies were conducted by X-MC and Y-JB. All authors contributed to the article and approved the submitted version.

## Funding

Supported by 1·3·5 project for disciplines of excellence Clinical Research Incubation Project, West China Hospital, Sichuan University (ZYJC18004).

## Conflict of interest

The authors declare that the research was conducted in the absence of any commercial or financial relationships that could be construed as a potential conflict of interest.

## Publisher’s note

All claims expressed in this article are solely those of the authors and do not necessarily represent those of their affiliated organizations, or those of the publisher, the editors and the reviewers. Any product that may be evaluated in this article, or claim that may be made by its manufacturer, is not guaranteed or endorsed by the publisher.

## Supplementary material

Supplementary Figure 1 The flow chart of experiment
