# Supplementary figures and images for: Case report: Dynamic antibody monitoring in a case of anti-recombinant human erythropoietin-mediated pure red cell aplasia with prolonged course after kidney transplantation
Source: Front Immunol. 2022 Nov 29;13:1049444. doi: 10.3389/fimmu.2022.1049444 (PMC9744924; doi:10.3389/fimmu.2022.1049444)

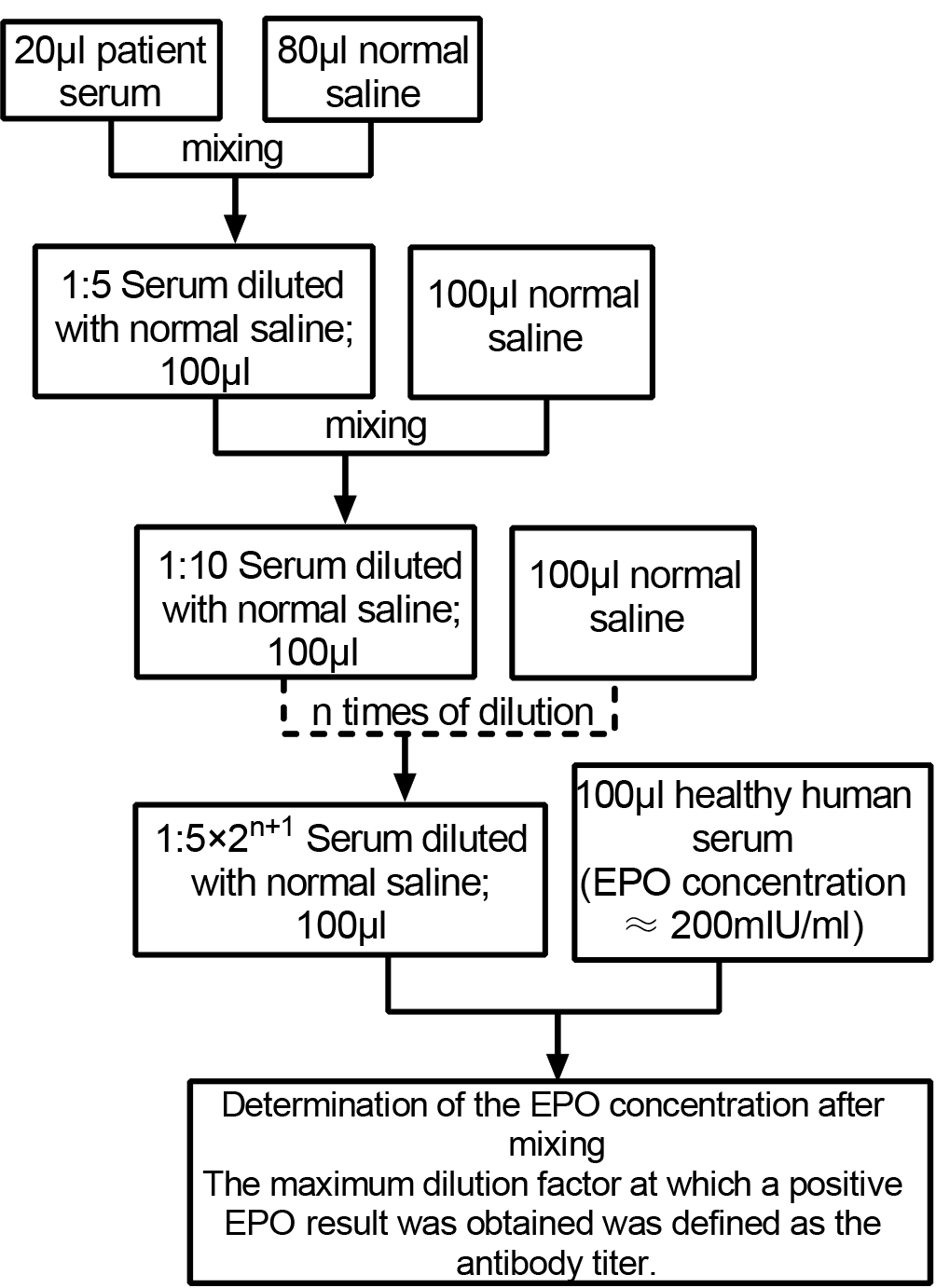

Supplement: Supplementary Figure 1 — The flow chart of experiment [file Image_1.tif]
